# OF VALUES AND P-VALUES: USING MIXED METHODS TO STUDY FAMILIES AND RELIGION IN THE LONGITUDINAL STUDY OF GENERATIONS

**DOI:** 10.1093/geroni/igz038.1533

**Published:** 2019-11-08

**Authors:** Merril D Silverstein

**Affiliations:** Syracuse University, Syracuse, New York, United States

## Abstract

In this presentation we discuss challenges and opportunities presented by the use of mixed methods to study continuity, change, and conflict related to religion—and the absence of religion—in multigenerational families. Discussion centers on the complementarity of quantitative and qualitative approaches, strategic decisions in tone and emphasis of the manuscript, and uncertainties in navigating the peer review process. An empirical example is provided to illustrates these points. We present an analysis that tracked patterns of religious change across three generations using survey data and in depth interviews emphasizing epistemological issues in knowledge generation.

